# Functional antibody responses targeting the Spike protein of SARS-CoV-2 Omicron XBB.1.5 in elderly nursing home residents following Wuhan-Hu-1-based mRNA booster vaccination

**DOI:** 10.1038/s41598-024-62874-7

**Published:** 2024-05-24

**Authors:** Ángela Sánchez-Simarro, Daniel Fernández-Soto, Brayan Grau, Eliseo Albert, Estela Giménez, Ana Isabel Avilés-Alía, Roberto Gozalbo-Rovira, Luciana Rusu, Beatriz Olea, Ron Geller, Hugh T. Reyburn, David Navarro

**Affiliations:** 1 Microbiology Service, Clinic University Hospital, INCLIVA Health Research Institute, Av. Blasco Ibáñez 17, 46010 Valencia, Spain; 2 Department of Immunology and Oncology, National Centre for Biotechnology, CNB-CSIC, Madrid, Spain; 3 Institute for Integrative Systems Biology (I2SysBio), Universitat de Valencia-CSIC, 46980 Valencia, Spain; 4 CIBER de Enfermedades Infecciosas, Instituto de Salud Carlos III, Madrid, Spain; 5 Department of Microbiology, School of Medicine, University of Valencia, Valencia, Spain

**Keywords:** Immunology, Microbiology

## Abstract

The immune effector mechanisms involved in protecting against severe COVID-19 infection in elderly nursing home residents following vaccination or natural infection are not well understood. Here, we measured SARS-CoV-2 Spike (S)-directed functional antibody responses, including neutralizing antibodies (NtAb) and antibody Fc-mediated NK cell activity (degranulation and IFNγ production), against the Wuhan-Hu-1, BA.4/5 (for NtAb), and Omicron XBB.1.5 variants in elderly nursing home residents (n = 39; median age, 91 years) before and following a third (pre- and post-3D) and a fourth (pre- and post-4D) mRNA COVID-19 vaccine dose. Both 3D and 4D boosted NtAb levels against both (sub)variants. Likewise, 3D and 4D increased the ability of sera to trigger both LAMP1- and IFNγ-producing NK cells, in particular against XBB.1.5. In contrast to NtAb titres, the frequencies of LAMP1- and IFNγ-producing NK cells activated by antibodies binding to Wuhan-Hu-1 and Omicron XBB.1.5 S were comparable at all testing times. Stronger functional antibody responses were observed in vaccine-experienced participants compared to vaccine-naïve at some testing times. These findings can contribute to identifying a reliable correlate of protection in elderly nursing home residents against severe COVID-19 and inform future vaccine strategies in this population group.

## Introduction

Adaptive B and T-cell immune responses elicited by vaccination, natural infection, or both are thought to play a pivotal role in protection against severe SARS-CoV-2 infection, although the contribution of each of these immunity arm contributes to this end remains to be precisely elucidated^1^. In the case of SARS-CoV-2, B cell immunity produces spike (S)-directed virus neutralizing antibodies (NtAb) and non-neutralizing antibodies mediating fragment crystallizable (Fc)-dependent effector functions that play a role in virus clearance^2–4^. The relevance of the latter functional antibody specificities has been elegantly demonstrated in a murine model, in which Fc-FcγR engagement and alveolar macrophages were shown to be required for vaccine-induced antibody-mediated protection against infection by antigenically diverse SARS-CoV-2 variants, including Omicron^5^. Importantly, NtAb targeting the S protein triggered by vaccination with Wuhan-Hu-1-based or newer COVID-19 vaccine platforms, as well as those generated by prior infection with older SARS-CoV-2 (sub)variants, display suboptimal efficacy against currently dominating Omicron subvariants (i.e. BA.2.86)^6–9^. However, recent findings indicate epitopes in the S gene that elicit Fc receptor antibody-mediated activation may be more conserved in emerging (sub)variants than those binding NtAb^5,9–15^.

COVID-19-related morbidity and mortality in elderly nursing home residents have dramatically decreased since the deployment of COVID-19 vaccines. This trend has continued following the emergence of Omicron subvariants following booster vaccination with the primary Wuhan-Hu-1-based vaccine or with (sub)variant-adapted vaccine platforms^16–18^. The immune effector mechanisms involved in protecting against severe COVID-19 infection in this population group remain poorly defined. In this sense, elderly nursing home residents with frailty and comorbidities frequently develop less robust anti-S antibody and NtAb responses following vaccination compared with seemingly healthy and younger counterparts, which also tend to wane faster^19–23^. In the current study, we measured S-directed functional antibody responses, including NtAb and Fc-mediated NK cell activity, against the ancestral SARS-CoV-2, Omicron BA.4/5 and Omicron XBB.1.5 variants in elderly nursing home residents following a third and a fourth mRNA COVID-19 vaccine dose.

## Methods

### Study population

A total of 39 elderly nursing home residents (31 females and 8 males; median age, 91 years; range, 66–103) were enrolled between August 2021 and October 2022. The Charlson comorbidity index (CCI)^24^, originally developed to predict one-year mortality was used to categorize the participants. It contains 19 issues including diabetes, congestive heart failure, peripheral vascular disease, chronic pulmonary disease, mild and severe liver disease, hemiplegia, renal disease, leukaemia, lymphoma, metastatic tumor, and acquired immunodeficiency syndrome (AIDS), each of which is weighted according to their potential influence on mortality. Severe CCI scores indexes are those ≥ 5. Participants in the current study had CCIs of 0–5 (n = 14), 6–10 (n = 23) and > 10 (n = 2). All participants had been previously evaluated for SARS-CoV-2 adaptive immunity following complete regular vaccination with Wuhan-Hu-1-based mRNA vaccines (22 with Comirnaty®, Pfizer/BioNTech and 17 with Spikevax®, Moderna)^25–29^. Whole blood was planned to be collected before and after one (pre-3D and post-3D, respectively) and two (pre-4D and post-4D, respectively) booster vaccine doses for the immunological analyses detailed below. The actual number of specimens from each participant that were tested by the different immunological assays described below is shown in Supplementary table 1. Some participants could not be sampled at a given time due to logistic reasons. Some participants from whom whole blood was collected could not be tested by a given assay due to insufficient volume. The first booster vaccine dose was homologous in all participants, whereas the second was heterologous in 17 participants (third dose with Spikevax® and fourth dose with Comirnaty®). Booster vaccine doses administered were non-adapted, Wuhan-Hu-1 based. The current study was carried out under the epidemiological surveillance competencies of the Valencia Government Health Department (Law 16/2003/May 28 on Cohesion and Quality of the National Health System, and Law 10/2014/ December 29 on Public Health of the Valencian Community), and was approved by the institutional ethical review board of the Foundation for the Promotion of Health and Biomedical Research in the Valencian Community (FISABIO) (Reference, 20210225/02/01). The requirement for informed consent was waived by the ethical review board of FISABIO. Likewise, according to local law and regulations, the publication of the data is exempt from the approval of a research ethics committee. Personal data from nursing homes and residents were deidentified and processed in accordance with European data protection regulations. All methods were performed in accordance with Declaration of Helsinki and the Belmont Report guidelines and regulations.

### SARS-CoV-2 immunoassays

SARS‐CoV‐2 total antibodies against the receptor binding domain (RBD) of Wuhan-Hu-1 S protein were quantified by the Roche Elecsys® Anti‐SARS‐CoV‐2 S test (Roche Diagnostics, Pleasanton, USA). Detection of anti-SARS-CoV-2 Nucleocapsid IgG in plasma specimens was performed using the Roche Elecsys® assay. NtAb targeting the S protein were quantified using a Green fluorescent protein (GFP)‐expressing vesicular stomatitis virus pseudotyped with the Wuhan‐Hu‐1^27^ and Omicron BA.4/5 (Addgene 186031), or XBB.1.5 (Addgene 196585) subvariants, as previously described^29^. The limit of detection of the assay was a reciprocal IC_50_ of 20 except for three samples in which the limit was 100 for Wuhan-Hu-1 and BA4.5. S due to limited sera availability. The antibody-mediated NK cell activation assay was based on that described by Chung et al. for HIV-1^30^, and later adapted to SARS-CoV-2^31^ and carried out as previously described^14^ with minor modifications. Briefly, purified, recombinant S proteins, corresponding to the SARS-CoV-2 Spike S1 + S2 trimer protein of either the D614G or Omicron XBB.1.5 variants (SinoBiological, Eschborn, Germany), were plated overnight in Thermo NUNC MaxiSorp 96-well plates at 3 μg/ml in borate buffered saline at 4ºC. After three washes with phosphate buffered saline-Tween (PBS-T) (PBS, 0.05% Tween 20), wells were blocked overnight in PBS 1% Casein (PBS-C) at 4ºC. Peripheral blood mononuclear cells (PBMC) were isolated from two healthy donors by centrifugation on Ficoll-Hypaque and rested overnight in RPMI 10% FCS supplemented with IL-12 (5 ng/ml) and IL-15 (1 ng/ml) (both from Peprotech, Thermofisher, Scientific, Waltham, USA). The next day, sera were diluted in PBS-C (normalized to anti-S trimeric IgG levels) added to the antigen-coated plates, and incubated at room temperature for 2 h. After three washes in PBS-T and three more in PBS, 50,000–100,000 PBMC were added per well in 100 μl of RPMI medium containing 10% FBS, 5 μg/ml Brefeldin A (Biolegend, San Diego, USA #420601), and 1 μg/ml anti-LAMP1 (lysosomal-associated membrane protein 1)-APC antibody (Biolegend, Clone H4A3). After a five-hour incubation at 37 °C and 5% CO2, cells were transferred to a U-bottom 96-well plate and stained with anti-CD3-FITC (Biolegend, Clone UCHT1) and anti-CD56-PC5 (Biolegend, Clone MEM-188) antibodies in benzo[a]pyrenebutyric acid (PBA) for 30’. After fixation for 5 min in 4% paraformaldehyde in PBS (PBF) at room temperature, cells were incubated with anti-IFNγ (Interferon-γ) PE/Cy7 (Biolegend, Clone 4S.B3) mAb in 0.25% Saponin. Stained cells were analysed using a Cytoflex flow cytometer (Beckman Coulter).

A previously described laboratory-developed assay was used for assessing SARS-CoV-2-S IgG avidity^32^. Briefly, SARS-CoV-2 RBD was produced in Sf9 insect cells infected with recombinant baculoviruses (Invitrogen; ThermoFischer). Following purification, the protein was concentrated to 5 mg/mL by ultrafiltration. Ninety-six well microplates were coated with RBD at 1 μg/mL. Serum samples were diluted 1:500 in PBS-T containing 1% bovine serum albumin and run in triplicate (mean values are reported). The plates were incubated with 1:5,000 dilution of horseradish peroxidase (HRP)-conjugated goat anti-human IgG (Jackson Laboratories, Bar Harbor, USA). After three washes with PBS-T, the binding was detected using SigmaFast OPD reagent (Sigma Aldrich, Saint Louis, USA) according to manufacturer’s recommendation. Color development was stopped with 3 M H_2_SO_4_ and read on a Multiskan FC plate reader at 492 nm. The cut-off discriminating between positive and negative sera was set as the mean absorbance of control sera plus three times the standard deviation. SARS-CoV-2 RBD IgG avidity index was calculated as the percentage of measured optical density (OD) in 6 M urea-treated wells relative to that in the untreated wells: AI (%) = OD of urea-treated well × 100/OD of non-urea-treated well. A positive-control (high avidity) specimen derived from a convalescent-phase serum from a COVID-19 patient (AI, 84%) was included on each ELISA plate. Specimens yielding AIs ranging between 101 and 120% were considered to have an AI of 100%.

### Statistical analyses

Frequency comparisons for categorical variables were carried out using Fisher’s exact test. Quantitative data are reported as medians and interquartile ranges (IQRs). Fold changes in antibody levels between testing times were calculated using non-log-transformed values. Differences between medians were compared using the Mann–Whitney U test for unpaired samples and the Wilcoxon test for paired specimens. The Spearman test was used for correlation analyses. Two‐sided exact *P* values are reported; a *P* value < 0.05 was considered statistically significant. The analyses were performed using SPSS version 20.0 (SPSS) and STATA 17.0 (StataCorp).

## Results

### SARS-CoV-2 vaccination and infection status of participants

The timing of booster COVID-19 vaccination and sampling across participants were as follows: pre-3D blood was collected a median of 181 days (range, 178–230) after completion of regular vaccination (two doses); post-3D blood was drawn a median of 17 days (range, 15–17) after receipt of 3D (median time between pre-3D and post-3D, 49 days; range, 20–73); pre-4D blood was obtained a median of 331 days (range, 198–339) post-3D and post-4D blood was collected a median of 112 days (range, 87–116) after 4D (median time elapsed between pre-4D and post-4D, 122 days; range, 116–127). SARS-CoV-2-experienced (Vac-ex) participants were those who had acquired SARS-CoV-2 infection; whereas SARS-CoV-2 naïve (Vac-n) were those who seemingly had not contracted SARS-CoV-2 infection. Categorization as Vac-ex or Vac-n was based upon the detection of anti-SARS-CoV-2-N IgG in plasma, a record of a positive RT-PCR result in nasopharyngeal specimens or both at the different time points of the study. At the time of study initiation (pre-3D), there were 27 Vac-n and 12 Vac-ex while only eight participants remained SARS-CoV-2-naïve at the end of the study period (post-4D). According to the dominant SARS-CoV-2 (sub)variant at a given time, 10 Vac-ex had been presumably infected by pre-Omicron (sub)variants, whereas the remaining 21 Vac-ex had been infected by Omicron (sub)variants (pre-XBB.1).

### Anti-SARS-CoV-2 RBD antibodies following receipt of COVID-19 vaccine booster doses

**Figure 1:**
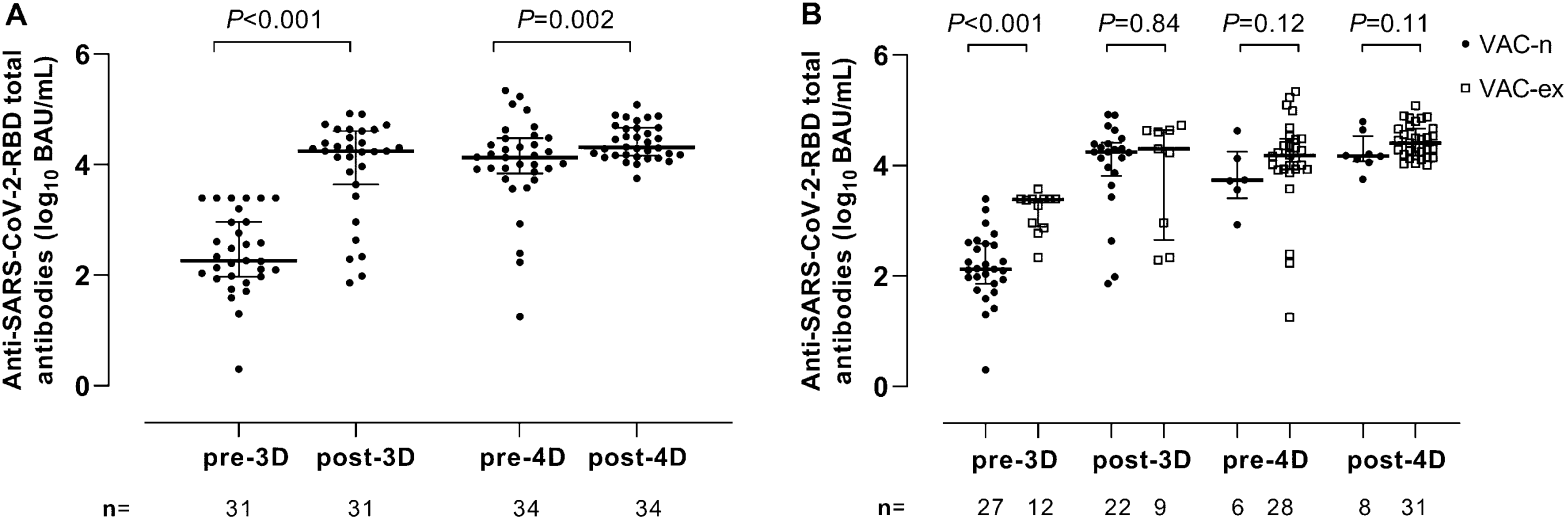
Anti-Receptor binding domain (RBD) total antibodies (in log_10_ in BAU/mL) in plasma from elderly nursing home residents. **A** antibody levels prior to and after one (pre-3D and post-3D, respectively) or two (pre-4D and post-4D, respectively) COVID-19 vaccine booster doses. **B** Antibody levels in vaccinated/SARS-CoV-2-experienced (Vac-ex) and vaccinated/SARS-CoV-2-naïve subjects (Vac-n) at the different sampling times. The number of plasma samples available for analyses at the different sampling times is shown. Bars indicate medians and IQRs. *P* values for differences are shown.

**Figure 2:**
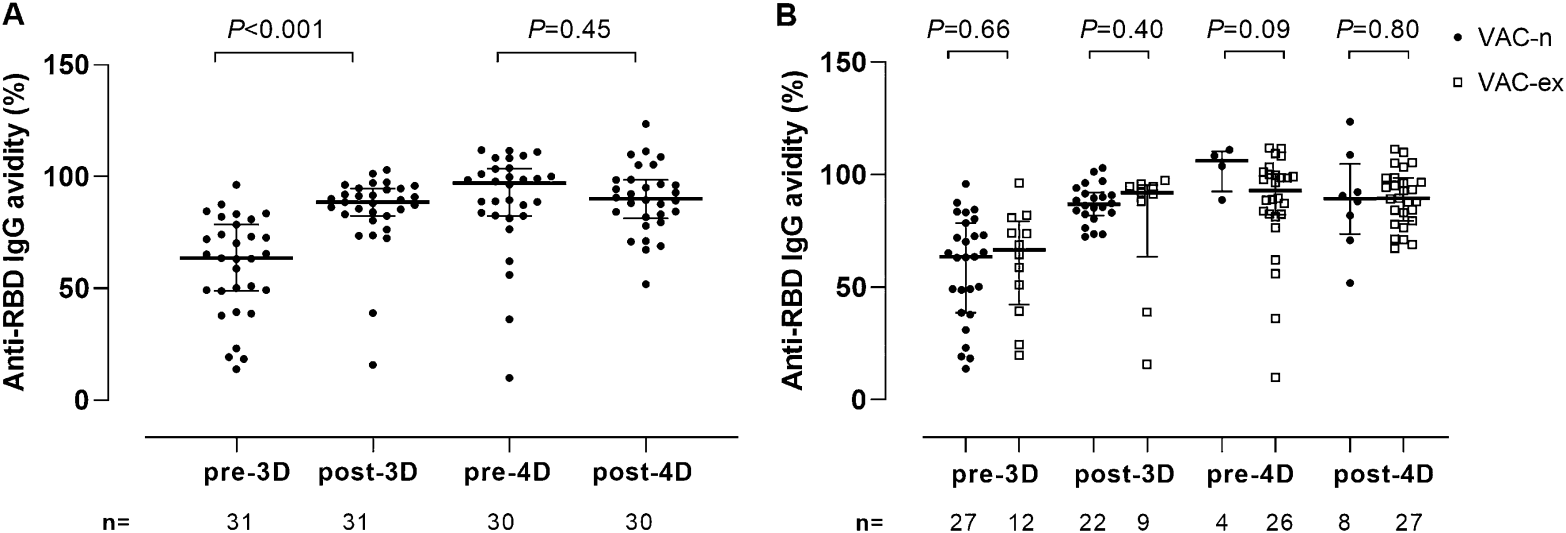
Anti-Receptor binding domain (RBD) IgG avidity in plasma from elderly nursing home residents. Total antibodies. **A** Anti-RBD IgG avidity indexes (%) before and after one (pre-3D and post-3D, respectively) or two (pre-4D and post-4D, respectively) COVID-19 vaccine booster doses. **B** Anti-RBD IgG avidity indexes (%) in vaccinated/SARS-CoV-2-experienced (Vac-ex) and vaccinated/SARS-CoV-2 naïve (Vac-n) at the different sampling times. The number of plasma samples available for analyses at the different sampling times is shown. Bars indicate medians and IQRs. *P* values for differences are shown.

All participants tested positive for anti-RBD total antibodies at all sampling times. Vaccination with 3D resulted in a 1.8-fold (*P* < 0.001) increase in anti-SARS-CoV-2-RBD total antibody levels compared with those pre-3D (median, 4.25 vs. 2.26 log_10_ BAU/ml) (Fig. 1A). Receipt of 4D also resulted in a significant increase of anti-RBD total antibody levels compared to pre-4D levels (4.31 vs. 4.12 log_10_ BAU/ml*; P* = 0.002) (Fig. 1A), this despite the time elapsed between pre-4D and post-4D was much longer than between pre-3D and post-3D. In both instances, the observed effect was irrespective of SARS-CoV-2 infection status. Anti-SARS-CoV-2-RBD total antibody levels pre-3D were significantly higher (*P* < 0.001) in Vac-ex than in Vac-n (3.38 vs. 2.12 log_10_ BAU/ml) but were comparable at the other sampling times (*P* ≥ 0.11; Fig. 1B). Since antibody avidity for a given antigen correlates with antibody functionality, we next sought to determine whether receipt of 3D or 4D had an impact on the anti-RBD IgG avidity index (AI). A number of specimens (4 pre-4D and 4 post-4D) yielded AIs above 120% due to low reactivity in the non-urea-treated wells; these samples could not be reanalized (lack of sufficient volume) and were excluded from the analyses below. We found that 3D drove a significant increase (*P* < 0.001) in the AI (pre-3D, median 63.6% vs. 88.5%) (Fig. 2A). This effect was noticed both in Vac-n (median 63.6% vs. 86.7%; *P* < 0.001) and Vac-ex (median 66.7% vs. 92%; *P* = 0.06) (Fig. 2B). Overall, anti-RBD IgG AIs measured pre-4D and post-4D were comparable (median 97.2% vs. 90.1%; *P* = 0.45) (Fig. 2A). At all sampling times, anti-RBD IgG AIs were not significantly different across Vac-n and Vac-ex (Fig. 2B).

### Neutralizing antibody responses against SARS-CoV-2 Wuhan-Hu-1, Omicron BA.4/5, and XBB.1.5 spike variants after COVID-19 vaccine boosters

**Figure 3:**
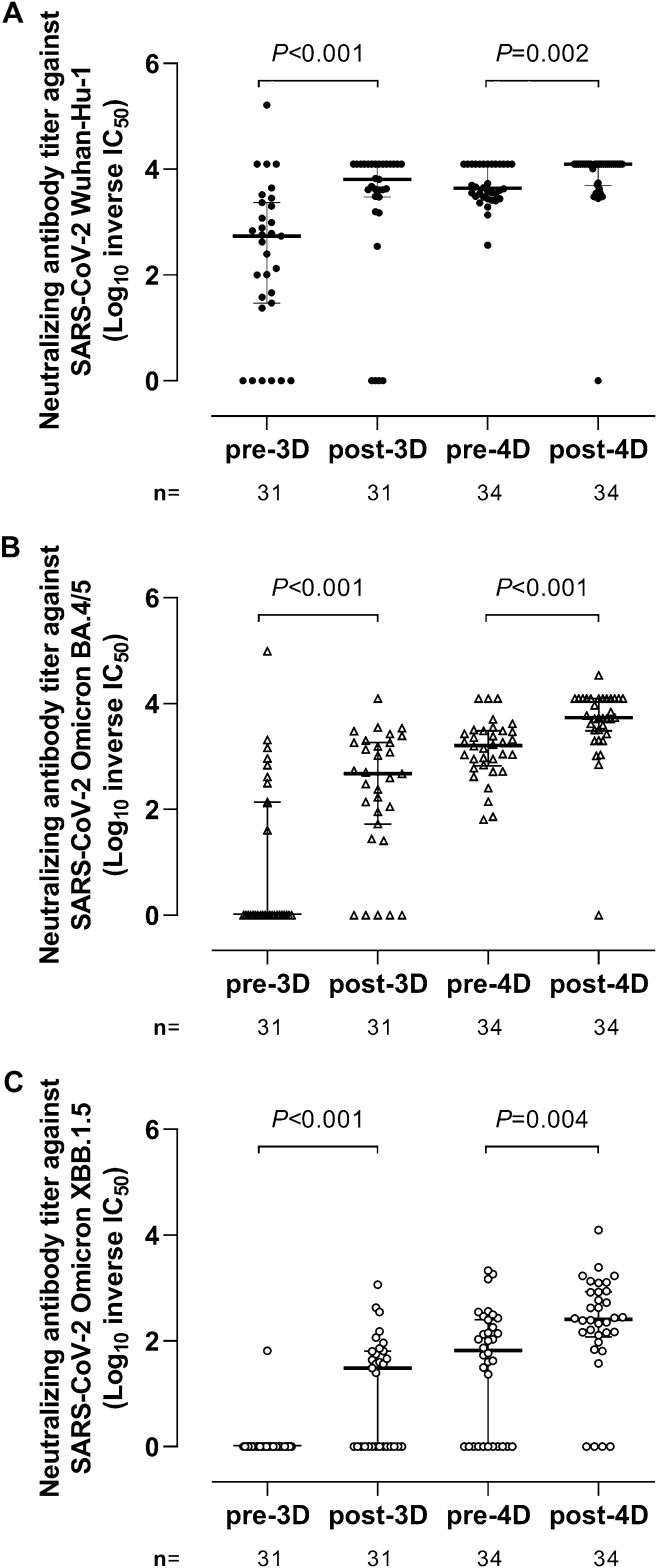
Neutralizing antibody titres (reciprocal IC_50_) against the spike protein of Wuhan-Hu-1 (**A**), Omicron BA.4/5 (**B**), and Omicron XBB.1.5 (**C**) in elderly nursing home residents before and after one (pre-3D and post-3D, respectively) or two (pre-4D and post-4D, respectively) COVID-19 vaccine booster doses. The number of plasma samples analysed at the different sampling times is shown. Bars indicate medians and IQRs. *P* values for differences are shown.

**Figure 4:**
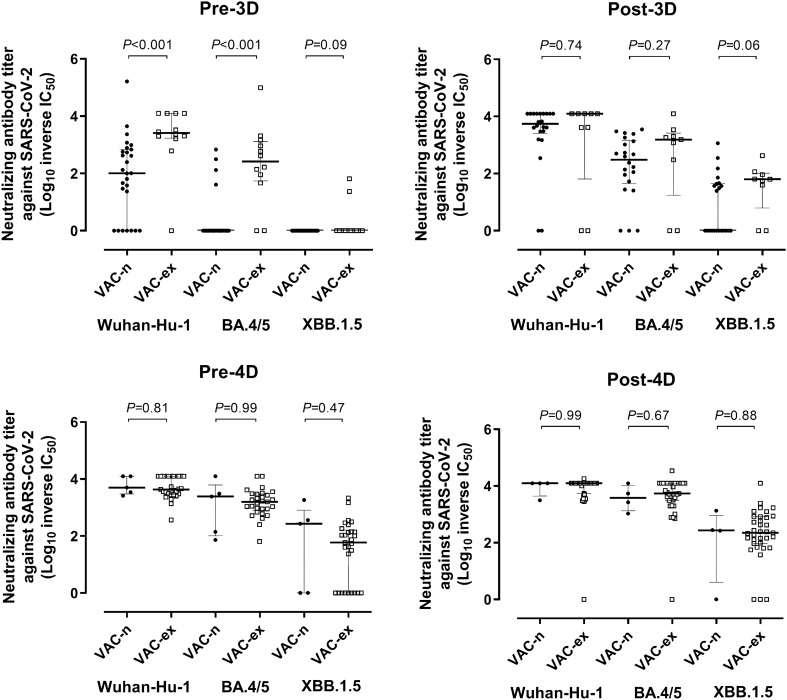
Neutralizing antibody titres (reciprocal IC_50_) against the spike protein of Wuhan-Hu-1, Omicron BA.4/5 , and Omicron XBB.1.5 in elderly nursing home residents before and after one (pre-3D and post-3D, respectively) or two (pre-4D and post-4D, respectively) COVID-19 vaccine booster doses, according to their SARS-CoV-2 infection status (experienced/Vac-ex or naïve/Vac-n). The number of plasma samples analysed at the different sampling times is shown. Bars indicate medians and IQRs. *P* values for differences are shown.

We next compared NtAb titres (reciprocal IC_50_) against SARS-CoV-2 Wuhan-Hu-1, Omicron BA.4/5, and Omicron XBB.1.5 S measured by a recombinant VSV-S-pseudotyped virus neutralization assay at the different study time points. The main observations derived from these experiments were as follows. First, overall (combining values at all sampling times), median NtAb titer against Wuhan-Hu-1 (4,297; IQR, 1,578–12,500) was significantly higher (*P* < 0.001) than that against Omicron BA.4/5 (1,043; IQR, 52–3,431) and Omicron XBB.1.5 (36.8; IQR, 0–181) (Fig. 3). Remarkably, only one participant had detectable NtAb against Omicron XBB.1.5 pre-3D and around half had NtAb against Omicron BA.4/5. Second, median NtAb titres against all SARS-CoV-2 (sub)variants significantly increased after 3D and 4D compared with pre-booster levels (Fig. 3). The NtAb titre increase post-3D varied from 11-fold against Wuhan-Hu-1 (Fig. 3A), nearly 500-fold against Omicron BA4/5 (Fig. 3B) and 30-fold against XBB.1.5 (Fig. 3C). An approximately three-fold increase in titers was observed against all (sub)variants following 4D (Fig. 3A–C); median NtAb titers post-4D were significantly higher than post-3D for all (sub)variants (*P* < 0.01). Third, at pre-3D, Vac-ex had significantly higher NtAb titers compared to Vac-n against Wuhan-Hu-1 and Omicron BA.4/5 (*P* < 0.001), but not against XBB.1.5 (*P* = 0.09) (Fig. 4, upper left panel); nevertheless, NtAb titers against all (sub)variants were not significantly different across Vac-ex and Vac-n at all other testing times.

### Antibody-dependent NK cell-mediated responses against SARS-CoV-2 Wuhan-Hu-1 and Omicron XBB.1.5 spike variants after vaccine boosting

**Figure 5:**
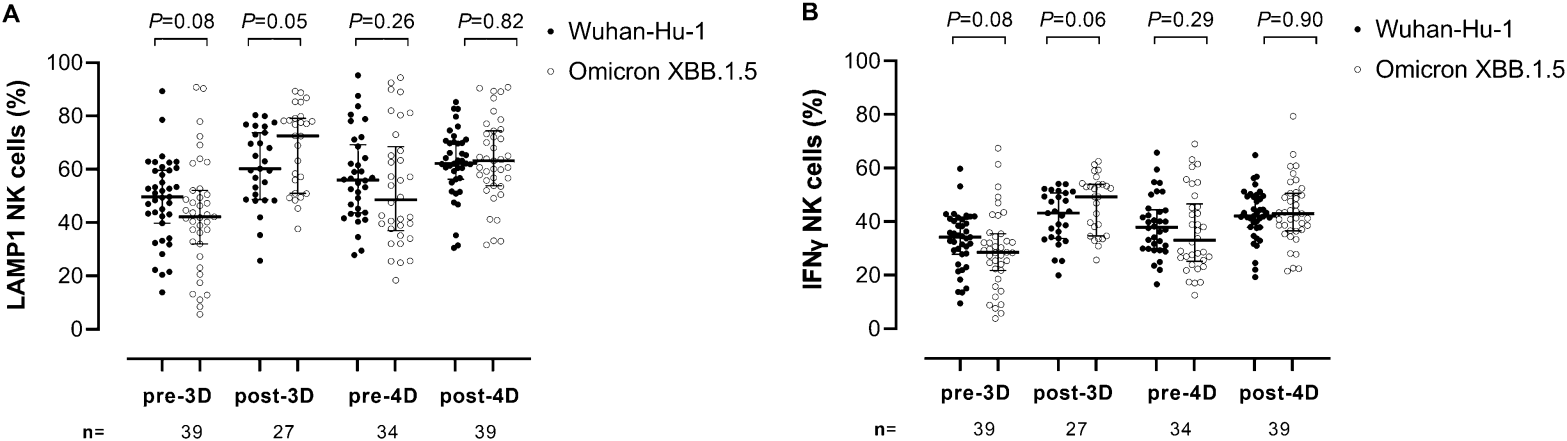
Comparison of antibody-dependent NK cell-mediated responses against SARS-CoV-2 Wuhan-Hu-1 and Omicron XBB.1.5 spike variants in elderly nursing home residents before and after one (pre-3D and post-3D, respectively) or two (pre-4D and post-4D, respectively) COVID-19 vaccine booster doses. Frequencies of LAMP1- (lysosomal-associated membrane protein 1) and IFN (interferon) γ-producing NK cells are shown in panels A and B, respectively. The number of participants analysed at the different sampling times is shown. Bars indicate medians and IQRs. *P* values for differences are shown.

**Figure 6:**
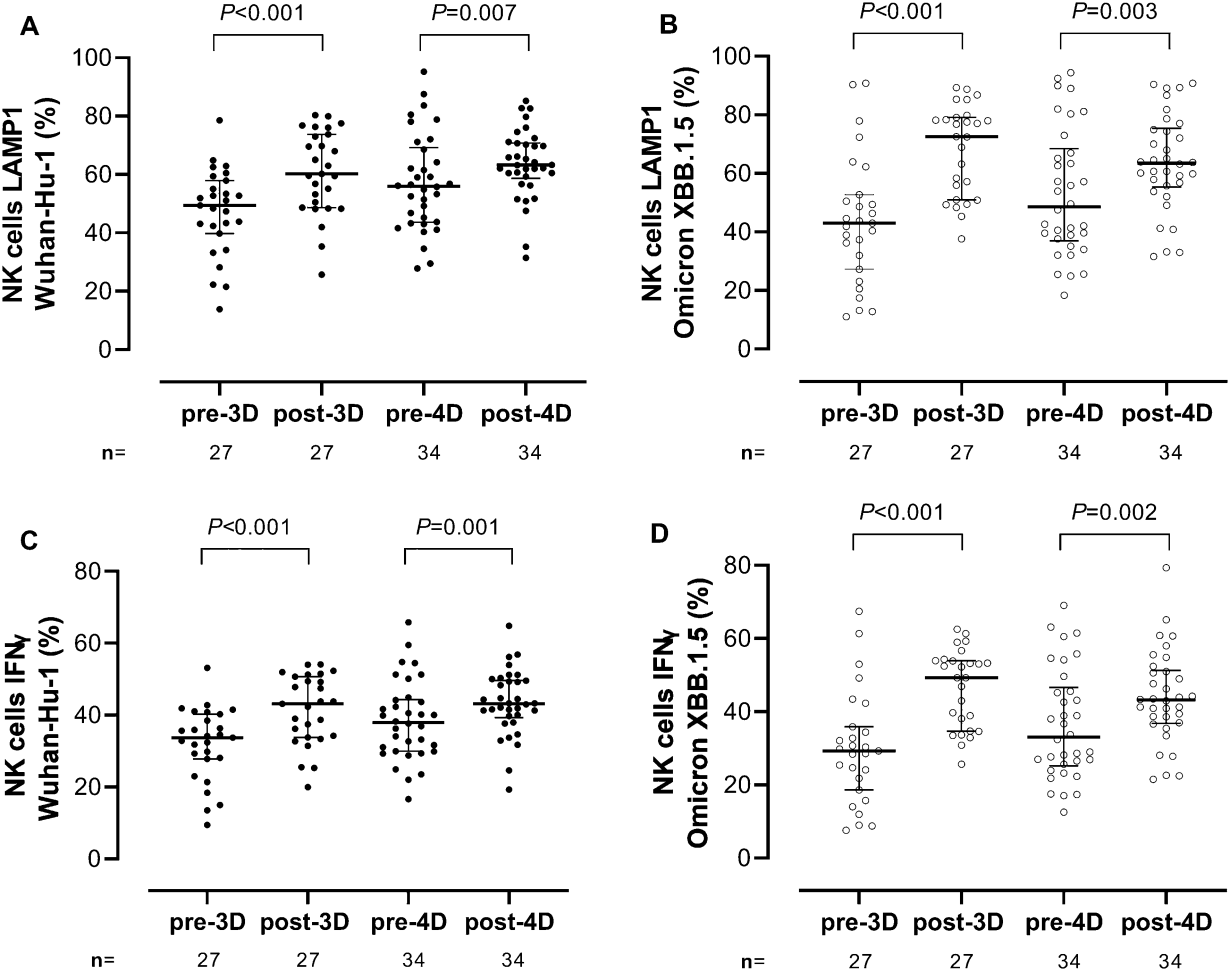
Impact of COVID-19 booster vaccination on antibody-dependent NK cell-mediated responses against SARS-CoV-2 Wuhan-Hu-1 and Omicron XBB.1.5 spike variants in elderly nursing home residents. The frequencies of LAMP1 (lysosomal-associated membrane protein 1)-producing NK cells against Wuhan-Hu-1 and Omicron XBB.1.5 are shown in (**A**) and (**B**), respectively. The frequencies of IFN (interferon) γ-producing NK cells against Wuhan-Hu-1 and Omicron XBB.1.5 are shown in (**C**) and (**D**), respectively. The number of participants analysed at the different sampling times is shown. Bars indicate medians and IQRs. *P* values for differences are shown.

**Figure 7:**
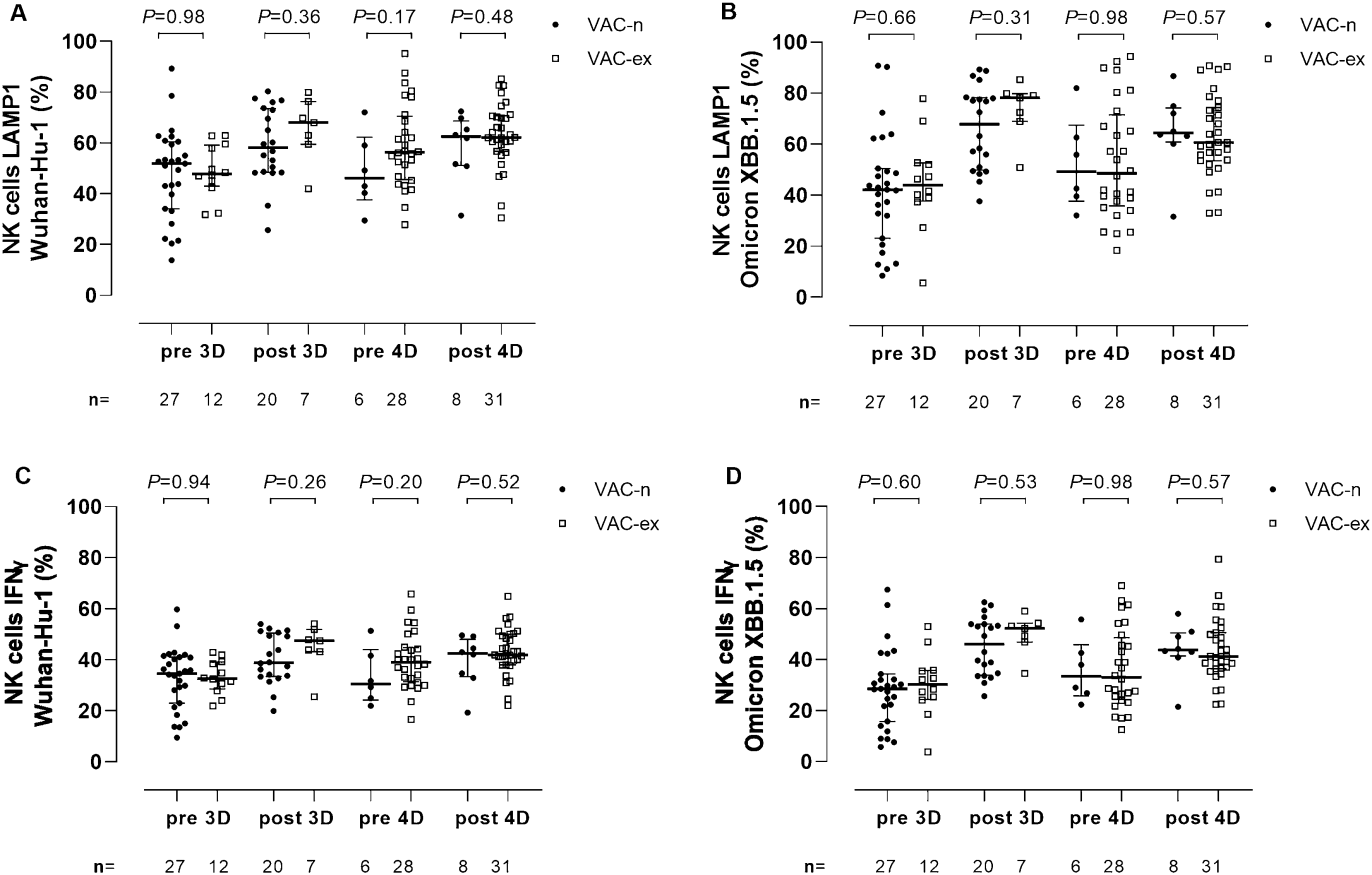
Antibody-dependent NK cell-mediated responses against SARS-CoV-2 Wuhan-Hu-1 and Omicron XBB.1.5 spike variants in elderly nursing home residents according to their SARS-CoV-2 infection status (experienced/Vac-ex or naïve/Vac-n). The frequencies of LAMP1- (lysosomal-associated membrane protein 1)-producing NK cells against Wuhan-Hu-1 and Omicron XBB.1.5 at the different testing times are shown in (**A**) and (**B**), respectively. The frequencies of IFN (interferon)γ-producing NK cells against Wuhan-Hu-1 and Omicron XBB.1.5 are shown in (**C**) and (**D**), respectively. The number of participants analysed at the different sampling times is shown. Bars indicate medians and IQRs. *P* values for differences are shown.

We next measured Fc-dependent S-directed antibody function using an antibody-dependent CD16A-mediated NK cell activity assay. PBMCs obtained from two healthy donors (donor 1 and donor 2) were used in all experiments, yielding similar results (not shown). Results obtained by using PBMCs from donor 1 are detailed below. All participants had detectable NK cell responses at all time points. Overall, the frequencies of LAMP1- and IFNγ- producing NK cells (Fig. 5A,B, respectively) did not differ significantly (*P* ≥ 0.5) depending on stimulation with Wuhan-Hu-1 or Omicron XBB.1.5 S at any testing times; nevertheless, at post-3D, a trend towards higher NK cell frequencies against XBB.1.5 compared to Wuhan-Hu-1was observed for both LAMP1 NK cells (72.5% vs. 60.2%; *P* = 0.05) and IFNγ NK cells (49.3% vs. 43.1%; *P* = 0.06) (Fig. 5A,B, respectively). Significantly higher frequencies of both LAMP1- or IFNγ-producing NK cells were observed post-3D and post-4D for both (sub)variants compared with pre-3D and pre-4D levels, respectively (Fig. 6, panels A-D). The increase in the frequency of LAMP1- and IFNγ-producing NK cells following the 3D and 4D boosters was more marked for XBB.1.5 than for Wuhan-Hu-1. When the frequency of functional NK cells activated by antibodies against Wuhan-Hu and Omicron XBB.1.5 was compared between Vac-ex and Vac-n participants, we found no significant differences at any sampling time for either LAMP1 (Fig. 7, panels A and B) or IFNγ-producing NK cells (Fig. 7, panels C and D).

### Correlation between Neutralizing antibody titres and antibody-dependent NK cell-mediated responses against SARS-CoV-2 Wuhan-Hu-1 and Omicron XBB.1.5

**Figure 8:**
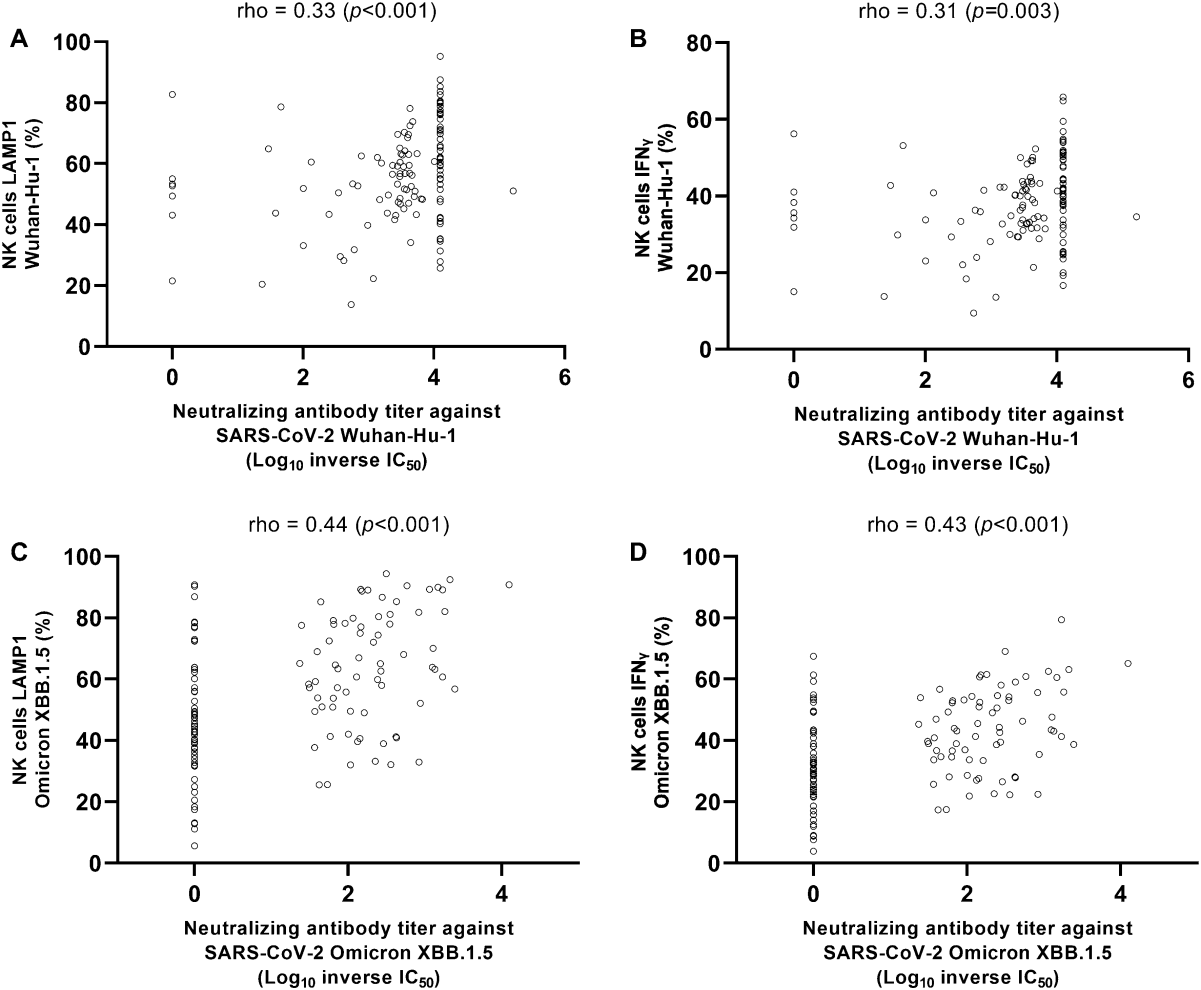
Correlation between Neutralizing antibody (NtAb) levels and frequencies of LAMP1- (lysosomal-associated membrane protein 1)-producing or (interferon)γ-producing NK cells against Wuhan-Hu-1 (**A** and **B**, respectively) and Omicron XBB.1.5 (**C** and **D**, respectively). Rho and P values by the Spearman Rank test are shown.

Overall, the correlation between NtAb titres and LAMP1- and IFNγ-producing NK cell frequencies was weak albeit significant against Wuhan-Hu-1 (Rho, 0.33; *P* < 0.001 and Rho, 0.31, P = 0.003, respectively) (Fig. 8 panels A and B) and moderate against Omicron XBB.1.5 (Rho, 0.44; *P* < 0.001; and Rho. 0.43, *P* < 0.001, respectively) (Fig. 8, panels C and D).

## Discussion

The underlying immune effector mechanisms providing protection against severe COVID-19 caused by the ancestral and emerging SARS-CoV-2 (sub)variants in elderly nursing home residents remain to be fully elucidated for both regular and booster vaccination. Here, we aimed to assess the overall impact of 3D and 4D booster doses on Wuhan-Hu-1 and Omicron XBB.1.5 S-reactive functional antibody levels, including NtAb and antibodies mediating NK cell activity through Fc. In addition, we sought to determine how these responses compared across SARS-CoV-2-experienced and naïve participants. Importantly, XBB.1.5 has been displaced by newly emerged Omicron subvariants such as BA.2.86; however, NtAb levels in sera from vaccinated individuals have been shown to display similar or even slightly higher efficiency against XBB.1.5 compared with current dominating variants, including BA.2.86, irrespective of whether a breakthrough infection with the latter (sub)variant had or had not occur^33,34^. Several major observations were made in the current study. First, both 3D and 4D boosted, to some extent, NtAb levels against both Wuhan-Hu-1 (and also anti-RBD total antibodies) and Omicron XBB.1.5. This effect was notably more marked following 3D as compared with 4D. Nevertheless, median NtAb titres post-4D were significantly higher than post-3D for all (sub)variants. Likewise, the increase in the frequency of both LAMP1- and IFNγ-producing NK cells following the 3D booster was more pronounced than after the 4D booster, particularly against XBB.1.5. Since antibody levels wane over time, the simplest explanation accounting for these findings is the longer time elapsed between vaccination and collection for the 4D as compared to the 3D; nevertheless, we cannot rule out that pre-existing SARS-CoV-2 specific antibodies limits humoral immunity boosting^35,36^. In keeping with the aforementioned observation, the anti-RBD IgG AI, which directly correlates with the level of antibody functionality^37^, significantly increased after the 3D but remained unchanged following the 4D. Second, while median NtAb titers against Wuhan-Hu-1 were significantly higher than those against Omicron XBB.1.5 at all testing times, the frequencies of LAMP1- and IFNγ- producing NK cells activated by plasma antibodies binding either to Wuhan-Hu-1 or Omicron XBB.1.5 S were comparable. Moreover, the correlation between NtAb titres and LAMP1- and IFNγ-producing NK cell frequencies against Omicron XBB.1.5 was moderate and weak against Wuhan-Hu-1. These findings reinforce the idea that antibodies mediating FcR activation either bind to more conserved S epitopes across SARS-CoV-2 (sub)variants than NtAb do or require a less stringent binding affinity for S epitopes than NtAb to be functionally effective^5,9–15^. Regardless of the underlying mechanistic explanation, antibodies mediating FcR NK cell activation seem to be less sensitive to SARS-CoV-2 escape by newly emerging variants than NtAb. This may have important implications regarding the COVID-19 vaccine and therapeutic moAb design. Third, a trend towards higher NtAb levels in Vac-ex compared with Vac-n after 3D, regardless of the SARS-CoV-2 (sub)variant, was observed pre-3D but not at other time points. A similar finding was previously reported by our group in apparently healthy individuals following regular COVID vaccination for NtAb binding the Wuhan-Hu-1 S and the Omicron BA.1 S protein^14^. In turn, the frequencies of both LAMP1- and IFNγ- producing NK cells activated by plasma antibodies were rather similar for Vac-Ex and Vac-n at all testing times. These data challenge the assumption that hybrid immunity provides stronger functional antibody responses than vaccination alone ^38–42^, at least in elderly nursing home residents receiving one or two booster vaccine doses. Fourth, our study provides further evidence of the increased ability of Omicron XBB.1.5 to escape from NtAb compared with preceding Omicron lineages, such as BA.4/5^33^.

The current study is not without limitations. First, its relatively limited sample size, in particular the small number of Vac-n after 3D, limits the robustness of some of the analyses performed. Second, as previously described^14^, a surrogate antibody‐mediated NK cell activation assay was used. Third, antibody‐mediated NK cell and NtAb responses against currently dominant Omicron subvariants (i.e., BA.2.86) were not evaluated. While these subvariants do not appear to outperform XBB.1.5 in terms of evasion from NtAb, despite incorporating additional mutations in the RBD domain^33,34^, their ability to escape antibodies mediating NK cell activity is unknown. Fourth, the SARS-CoV-2 (subvariant) infecting the participants was inferred, based upon the timing of infection, but not proven by genome sequencing approaches. This precluded meaningful analyses addressing the potential impact of the infecting (sub)variant on the strength of the functional antibody responses measured. Moreover, we could not ascertain whether participants were infected one or more times by different lineages. Furthermore, categorization as Vac-ex or Vac-n on the basis of the presence or absence of detectable anti-SARS-CoV-2 N antibodies, respectively, may be erroneous due to waning of such antibody specificities. Fifth, due to logistic constraints, FC-mediated NK cell responses against Omicron BA.4/5 were not assessed. Sixth, there was no control group. Finally, we wish to highlight a major limitation of the study; that is the is the different time elapsed between receipt of 3D and 4D and post-3D and post-4D immunological testing. This precluded to properly compare the impact of both booster vaccine doses on functional antibody titers. Unfortunately, logistic issues precluded testing within an identical timeframe. In summary, our data revealed that that both 3D and 4D boostered functional antibody responses and further stressed the fact that NK cell Fc-dependent functional activity against Omicron XBB.1.5 is relatively conserved in fully vaccinated participants boosted with Wuhan-Hu-1-based mRNA vaccines. The data presented herein can contribute to identifying a reliable correlate of protection in elderly nursing home residents, potentially helping to recognize individuals at particular risk of severe COVID-19 and inform future vaccine strategies in this population group.

### Supplementary Information

Supplementary Information.

## Data Availability

The data that support the findings of this study are available from the corresponding author upon reasonable request.

## References

[1] Goldblatt D, Alter G, Crotty S, Plotkin SA (2022). Correlates of protection against SARS-CoV-2 infection and COVID-19 disease. Immunol. Rev..

[2] Qi H, Liu B, Wang X, Zhang L (2022). The humoral response and antibodies against SARS-CoV-2 infection. Nat Immunol..

[3] Gruell H, Vanshylla K, Weber T, Barnes CO, Kreer C, Klein F (2022). Antibody-mediated neutralization of SARS-CoV-2. Immunity..

[4] Zhang A, Stacey HD, D'Agostino MR, Tugg Y, Marzok A, Miller MS (2023). Beyond neutralization: Fc-dependent antibody effector functions in SARS-CoV-2 infection. Nat. Rev. Immunol..

[5] Mackin SR (2023). Fc-γR-dependent antibody effector functions are required for vaccine-mediated protection against antigen-shifted variants of SARS-CoV-2. Nat Microbiol..

[6] Shrestha LB, Foster C, Rawlinson W, Tedla N, Bull RA (2022). Evolution of the SARS-CoV-2 omicron variants BA.1 to BA.5: Implications for immune escape and transmission. Rev. Med. Virol..

[7] Wang Q (2023). Alarming antibody evasion properties of rising SARS-CoV-2 BQ and XBB subvariants. Cell..

[8] Pather S (2023). SARS-CoV-2 Omicron variants: burden of disease, impact on vaccine effectiveness and need for variant-adapted vaccines. Front. Immunol..

[9] Grunst MW, Uchil PD (2022). Fc effector cross-reactivity: A hidden arsenal against SARS-CoV-2’s evasive maneuvering. Cell Rep. Med..

[10] Richardson SI (2022). SARS-CoV-2 Beta and Delta variants trigger Fc effector function with increased cross-reactivity. Cell Rep. Med..

[11] Kaplonek P (2022). mRNA-1273 vaccine-induced antibodies maintain Fc effector functions across SARS-CoV-2 variants of concern. Immunity..

[12] Richardson SI (2022). SARS-CoV-2 Omicron triggers cross-reactive neutralization and Fc effector functions in previously vaccinated, but not unvaccinated, individuals. Cell Host Microbe..

[13] Richardson SI (2023). Antibody-dependent cellular cytotoxicity against SARS-CoV-2 Omicron sub-lineages is reduced in convalescent sera regardless of infecting variant. Cell Rep. Med..

[14] Albert E (2023). Antibody-dependent NK-cell and neutralizing antibody responses against the Spike protein of Wuhan-Hu-1 and Omicron BA.1 SARS-CoV-2 variants in vaccinated experienced and vaccinated naïve individuals. J. Med. Virol..

[15] Bartsch YC (2022). Omicron variant Spike-specific antibody binding and Fc activity are preserved in recipients of mRNA or inactivated COVID-19 vaccines. Sci. Transl. Med..

[16] Nordström P, Ballin M, Nordström A (2022). Effectiveness of a fourth dose of mRNA COVID-19 vaccine against all-cause mortality in long-term care facility residents and in the oldest old: a nationwide, retrospective cohort study in Sweden. Lancet Reg. Health Eur..

[17] Blom K (2023). SARS-CoV-2-related mortality decrease in nursing home residents given multiple COVID-19 boosters. Lancet Infect. Dis..

[18] Muhsen K (2022). Association of BNT162b2 vaccine third dose receipt with incidence of SARS-CoV-2 infection, COVID-19–Related Hospitalization, and Death Among Residents of Long-term Care Facilities, August to October 2021. JAMA Netw. Open..

[19] Collier DA (2021). Age-related immune response heterogeneity to SARS-CoV-2 vaccine BNT162b2. Nature..

[20] Canaday DH (2022). COVID-19 vaccine booster dose needed to achieve Omicron-specific neutralisation in nursing home residents. eBioMedicine..

[21] Tober-Lau P (2021). Long-term immunogenicity of BNT162b2 vaccination in older people and younger health-care workers. Lancet Respir. Med..

[22] Renia L (2022). Lower vaccine-acquired immunity in the elderly population following two-dose BNT162b2 vaccination is alleviated by a third vaccine dose. Nat. Commun..

[23] Pannus P (2023). Third dose of COVID-19 mRNA vaccine closes the gap in immune response between naïve nursing home residents and healthy adults. Vaccine..

[24] Charlson ME (1987). A new method of classifying prognostic comorbidity in longitudinal studies: development and validation. J. Chronic. Dis..

[25] Torres I (2021). B- and T-cell immune responses elicited by the Comirnaty® COVID-19 vaccine in nursing-home residents. Clin. Microbiol. Infect..

[26] Albert E (2022). Immunological response against SARS-CoV-2 following full-dose administration of Comirnaty® COVID-19 vaccine in nursing home residents. Clin. Microbiol. Infect..

[27] Giménez E (2022). Evolution of SARS-CoV-2 immune responses in nursing home residents following full dose of the Comirnaty® COVID-19 vaccine. J. Infect..

[28] Giménez E (2022). Severe acute respiratory syndrome coronavirus 2 adaptive immunity in nursing home residents following a third dose of the comirnaty coronavirus disease 2019 vaccine. Clin. Infect. Dis..

[29] Sánchez-Sendra B (2022). Neutralizing antibodies against SARS-CoV-2 variants of concern elicited by the Comirnaty COVID-19 vaccine in nursing home residents. Sci. Rep..

[30] Chung AW (2015). Dissecting polyclonal vaccine-induced humoral immunity against HIV using systems serology. Cell..

[31] Zohar T (2020). Compromised humoral functional evolution tracks with SARS-CoV-2 mortality. Cell..

[32] Gozalbo-Rovira R (2020). SARS-CoV-2 antibodies, serum inflammatory biomarkers and clinical severity of hospitalized COVID-19 patients. J. Clin. Virol..

[33] Lasrado N (2023). Neutralization escape by SARS-CoV-2 Omicron subvariant BA.2.86. Vaccine..

[34] Sheward DJ (2023). Sensitivity of the SARS-CoV-2 BA.2.86 variant to prevailing neutralising antibody responses. Lancet Infect. Dis..

[35] Zhang Y, Meyer-Hermann M, George LA (2013). Germinal center B cells govern their own fate via antibody feedback. J. Exp. Med..

[36] Tas JMJ, Koo JH, Lin YC (2022). Antibodies from primary humoral responses modulate the recruitment of naive B cells during secondary responses. Immunity..

[37] Oostindie SC, Lazar GA, Schuurman J, Parren PWHI (2022). Avidity in antibody effector functions and biotherapeutic drug design. Nat. Rev. Drug Discov..

[38] Goldberg Y, Mandel M, Bar-On YM (2021). Waning immunity after the BNT162b2 vaccine in Israel. N. Engl. J. Med..

[39] Ebinger JE, Fert-Bober J, Printsev I (2021). Antibody responses to the BNT162b2 mRNA vaccine in individuals previously infected with SARS-CoV-2. Nat. Med..

[40] Stamatatos L, Czartoski J, Wan YH (2021). mRNA vaccination boosts cross-variant neutralizing antibodies elicited by SARS-CoV-2 infection. Science..

[41] Goldberg Y, Mandel M, Bar-On YM (2022). Protection and waning of natural and hybrid immunity to SARS-CoV-2. N. Engl. J. Med..

[42] Epsi NJ, Richard SA, Lindholm DA (2023). Understanding “hybrid immunity”: comparison and predictors of humoral immune responses to severe acute respiratory syndrome coronavirus 2 infection (SARS-CoV-2) and coronavirus disease 2019 (COVID-19) vaccines. Clin. Infect. Dis..

