# Supplementary material for: Functional antibody responses targeting the Spike protein of SARS-CoV-2 Omicron XBB.1.5 in elderly nursing home residents following Wuhan-Hu-1-based mRNA booster vaccination
Source: Sci Rep. 2024 May 24;14:11896. doi: 10.1038/s41598-024-62874-7 (PMC11126592; doi:10.1038/s41598-024-62874-7)
Supplement: Supplementary file 1 — Supplementary Information. [file 41598_2024_62874_MOESM1_ESM.docx]

| **Supplementary Table 1. Plasma specimens available from each participant for immunological analyses** | | | | | | | | | | | | |
| --- | --- | --- | --- | --- | --- | --- | --- | --- | --- | --- | --- | --- |
| **Patient number** | **Samples available for Anti-SARS-CoV-2-RBD and Anti-RBD IgG avidity testing** | | | | **Samples available for neutralizing antibody against Wuhan-Hu-1, Omicron BA.4/5 and XBB.1.5 testing** | | | | **Samples available for antibody-mediated NK cells producing LAMP1 and IFN- γ testing** | | | |
|  | Pre-3D | Post-3D | Pre-4D | Post-4D | Pre-3D | Post-3D | Pre-4D | Post-4D | Pre-3D | Post-3D | Pre-4D | Post-4D |
| 1 | x | x | x | x | x | x | x | x | x | x | x | x |
| 2 | x |  | x | x | x |  | x | x | x |  | x | x |
| 3 | x | x | x | x | x | x | x | x | x | x | x | x |
| 4 | x | x | x | x | x | x | x | x | x | x | x | x |
| 5 | x | x | x | x | x | x | x | x | x | x | x | x |
| 6 | x | x | x | x | x | x | x | x | x | x | x | x |
| 7 | x | x | x | x | x | x | x | x | x | x | x | x |
| 8 | x |  | x | x | x |  | x | x | x |  | x | x |
| 9 | x | x | x | x | x | x | x | x | x | x | x | x |
| 10 | x | x | x | x | x | x | x | x | x | x | x | x |
| 11 | x | x | x | x | x | x | x | x | x | x | x | x |
| 12 | x | x | x | x | x | x | x | x | x | x | x | x |
| 13 | x | x | x | x | x | x | x | x | x | x | x | x |
| 14 | x | x | x | x | x | x | x | x | x | x | x | x |
| 15 | x | x | x | x | x | x | x | x | x | x | x | x |
| 16 | x | x | x | x | x | x | x | x | x | x | x | x |
| 17 | x | x | x | x | x | x | x | x | x | x | x | x |
| 18 | x | x | x | x | x | x | x | x | x |  | x | x |
| 19 | x | x | x | x | x | x | x | x | x | x | x | x |
| 20 | x | x |  | x | x | x |  | x | x | x |  | x |
| 21 | x |  | x | x | x |  | x | x | x |  | x | x |
| 22 | x | x | x | x | x | x | x | x | x | x | x | x |
| 23 | x | x |  | x | x | x |  | x | x | x |  | x |
| 24 | x | x |  | x | x | x |  | x | x |  |  | x |
| 25 | x |  | x | x | x |  | x | x | x |  | x | x |
| 26 | x | x | x | x | x | x | x | x | x | x | x | x |
| 27 | x | x |  | x | x | x |  | x | x |  |  | x |
| 28 | x | x | x | x | x | x | x | x | x | x | x | x |
| 29 | x | x | x | x | x | x | x | x | x | x | x | x |
| 30 | x | x | x | x | x | x | x | x | x | x | x | x |
| 31 | x | x | x | x | x | x | x | x | x | x | x | x |
| 32 | x | x | x | x | x | x | x | x | x |  | x | x |
| 33 | x |  | x | x | x |  | x | x | x | x | x | x |
| 34 | x | x | x | x | x | x | x | x | x |  | x | x |
| 35 | x |  | x | x | x |  | x | x | x | x | x | x |
| 36 | x | x | x | x | x | x | x | x | x |  | x | x |
| 37 | x |  | x | x | x |  | x | x | x | x | x | x |
| 38 | x | x | x | x | x | x | x | x | x |  | x | x |
| 39 | x |  |  | x | x |  |  | x | x |  |  | x |
|  | n=39 | N=31 | n=34 | n=39 | n=39 | n=31 | n=34 | n=39 | n=39 | n=27 | n=34 | n=39 |
| LAMP1, lysosomal-associated membrane protein 1; IFN- γ, Interefron-gamma; Pre-3D and post-3D, prior and after the first vaccine booster dose (3D); Pre-4D and post-4D, prior and after the second vaccine booster dose (4D); RBD, receptor binding domain; X, available for the analysis indicated. | | | | | | | | | | | | |
|  |  |  |  |  |  |  |  |  |  |  |  |  |
